# Specularly-Reflected Wave Guidance of Terahertz Plasmonic Metamaterial Based on the Metal-Wire-Woven Hole Arrays: Functional Design and Application of Transmission Spectral Dips

**DOI:** 10.3390/ma16124463

**Published:** 2023-06-19

**Authors:** Borwen You, Ryuji Iwasa, Po-Lun Chen, Tun-Yao Hung, Chih-Feng Huang, Chin-Ping Yu, Hsin-Ying Lee

**Affiliations:** 1 Department of Physics, National Changhua University of Education, No. 1 Jinde Road, Changhua 500207, Taiwan; 2 Department of Applied Physics, Faculty of Pure and Applied Sciences, University of Tsukuba, Tennodai 1-1-1, Tsukuba 305-8573, Ibaraki, Japan; iwasa.ryuji.abc@gmail.com; 3 Department of Photonics, National Cheng Kung University, No. 1 University Road, Tainan 70101, Taiwan; r09941156@ntu.edu.tw (P.-L.C.); hylee@ee.ncku.edu.tw (H.-Y.L.); 4 Department of Photonics, National Sun Yat-Sen University, Kaohsiung 80424, Taiwan; tunyaohung.ee09@nycu.edu.tw (T.-Y.H.); cpyu@faculty.nsysu.edu.tw (C.-P.Y.); 5 Department of Chemical Engineering, i-Center for Advanced Science and Technology (iCAST), National Chung Hsing University, 145 Xingda Road, South District, Taichung 40227, Taiwan; huangcf@dragon.nchu.edu.tw

**Keywords:** terahertz radiation, artificial material, spectroscopy, aperture antennas, electromagnetic scattering by periodic structures, high-pass filters, submillimeter wave waveguides, time domain measurements, frequency domain analysis

## Abstract

Terahertz (THz) plasmonic metamaterial, based on a metal-wire-woven hole array (MWW-HA), is investigated for the distinct power depletion in the transmittance spectrum of 0.1–2 THz, including the reflected waves from metal holes and woven metal wires. Woven metal wires have four orders of power depletion, which perform sharp dips in a transmittance spectrum. However, only the first-order dip at the metal–hole–reflection band dominates specular reflection with a phase retardation of approximately π. The optical path length and metal surface conductivity are modified to study MWW-HA specular reflection. This experimental modification shows that the first order of MWW-HA power depletion is sustainable and sensitively correlated with a bending angle of the woven metal wire. Specularly reflected THz waves are successfully presented in hollow-core pipe wave guidance specified from MWW-HA pipe wall reflectivity.

## 1. Introduction

Terahertz (THz) waves, in the frequency range of 0.1–2 THz, exactly cover the millimeter and sub-millimeter electromagnetic (EM) waves that act as radiation in the new generation of wireless communication [1,2]. The investigation of the THz transceiver becomes critical because the short broadcast distance of THz radiation resembles that of free space optics, which is in contrast to that of microwave radiation [3,4]. Signal transportation among THz transceivers can improve the low diffraction efficiency of THz communication in a complex space, which is composed of many sharp shadows of building walls, people, and certain obstacles to block THz EM signals [1]. Efficiently controlling or manipulating the amplitude or phase of THz radiation through solid-state media is, thus, critical for achieving the signal connection through THz transceivers. Hollow core pipe waveguides, with inner reflection configurations of single plastic sheets [5], metal-coated surfaces [6], and various microstructural [7,8,9] rings, have distinct advantages when performing as wireless telecom transceivers, including a large acceptance angle to receive THz emission, low waveguide loss, uniform modal field, and flexibly directional operation.

The meta-surfaces and metamaterials with subwavelength-scaled and periodic patterns facilitate THz surface-cumulated EM waves on discontinuous metal surfaces, typically called plasmonic metamaterials, with artificial plasmonic frequencies that pass and reflect THz waves [10], respectively, in the high and low-frequency bands. For the development of THz plasmonic metamaterials as one inner reflector layer of the pipe waveguide materials, the simple and easily fabricated plasmonic metamaterials, such as the circular, square, and slot–hole arrays [11], are crucial due to the application purpose of a large-area operation. These subwavelength-scaled metal patterns are normally perforated on metal sheets, but the propagation constants in THz wave phases cannot be controlled from the periodically perforated plane, i.e., the planar configurations. Currently, the meta-mirror device has been presented, but two layers of metamaterials with one specified air space are requested for the performance of specular reflection [12], i.e., the optimally reflective performance. Furthermore, the periodic metal patterns on the two layers of metamaterials should be supported by rigid substrates that are limited to deformation as pipe walls [12] for the long-distance or large-area propagation of THz waves.

In this paper, the THz plasmonic metamaterial, based on a metal-wire woven hole array (MWW-HA), is characterized for 0.1–2 THz wave transmission based on the geometry of periodically corrugated metal wires and the integration of polymer dielectric, conductive, and insulator layers. After this spectral and structural investigation of the MWW-HA bulk material, the presented transmission spectral dips, in the broad bandwidth of 1 or 2 THz, can further be designed for the specularly reflected function and one waveguide application. In the experimental results, the MWW-HA is robust and flexible, and it can be deformed as one large core pipe waveguide, with scale of approximately a centimeter, to receive THz waves and zigzag reflect through the pipe wall for a long distance. The specular reflection is the MWW-HA structural criterion used to approach the low-loss performance of a zigzag reflection. The MWW-HA pipe waveguide can, potentially, be used as a transceiver in future wireless communication to both receive and transmit THz radiation at indicated channel spaces to prevent the propagation loss of obstacles.

## 2. Materials and Methods

### 2.1. Configuration of an MWW-HA

**Figure 1:**
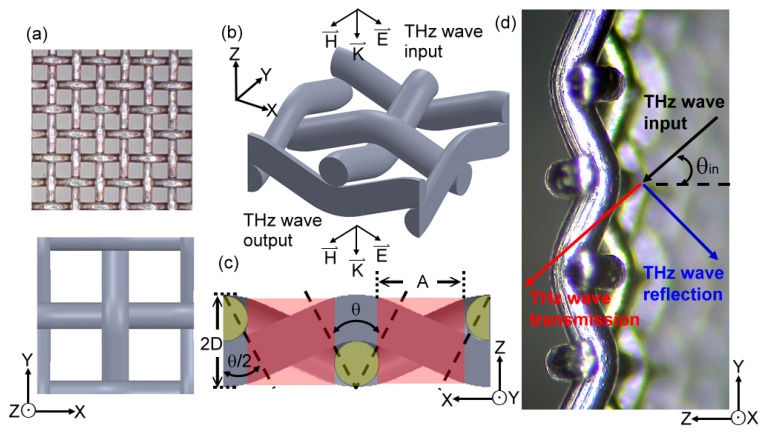
(**a**) Top, (**b**) three-dimensional, and (**c**,**d**) side views of the MWW-HA configuration.

The THz plasmonic metamaterial, based on MWW-HAs, is presented in Figure 1, including the mechanical drawings and photographs. Figure 1a illustrates the top-view configuration that indicates the square and periodic hole array in the X–Y plane, with a pore width of *A* and a metal-wire width of *D*. The three-dimensional and side-view configurations of MWW-HA are shown in Figure 1b,c, respectively, presenting the mechanical structure of a woven metal wire. The bending angle *θ* of a woven metal wire critically performs on the bases of the pore width (*A*) and structural thickness (2 × *D*) parameters as schematically illustrated in the side-view drawing of the X–Z plane. The three-dimensional view of the mechanical drawing in Figure 1b shows two pairs of metal wires weaving an MWW-HA unit. A pair of woven metal wires along the X or Y axis is oppositely bent toward the +Z and –Z directions. Thus, the X and Y-axial pairs of woven metal wires construct four square holes within an MWW-HA unit, whose space is marked with red in the side view configuration [Figure 1c]. The photograph of Figure 1d presents the side-viewed MWW-HA, which is woven by stainless steel metal wires in this study. The corresponding *A*, *D*, and *θ* used in this paper are listed in Table 1. Those MWW-HAs with structural numbers from 1 to 6 (Table 1) are normal and available from hardware stores without any fabrication process. To characterize the MWW-HAs in THz spectroscopy, the THz waves are normally input on MWW-HAs [Figure 1d, *θ_in_* = 0°].

### 2.2. Integrations of Dielectric and Conductive Layers

**Figure 2:**
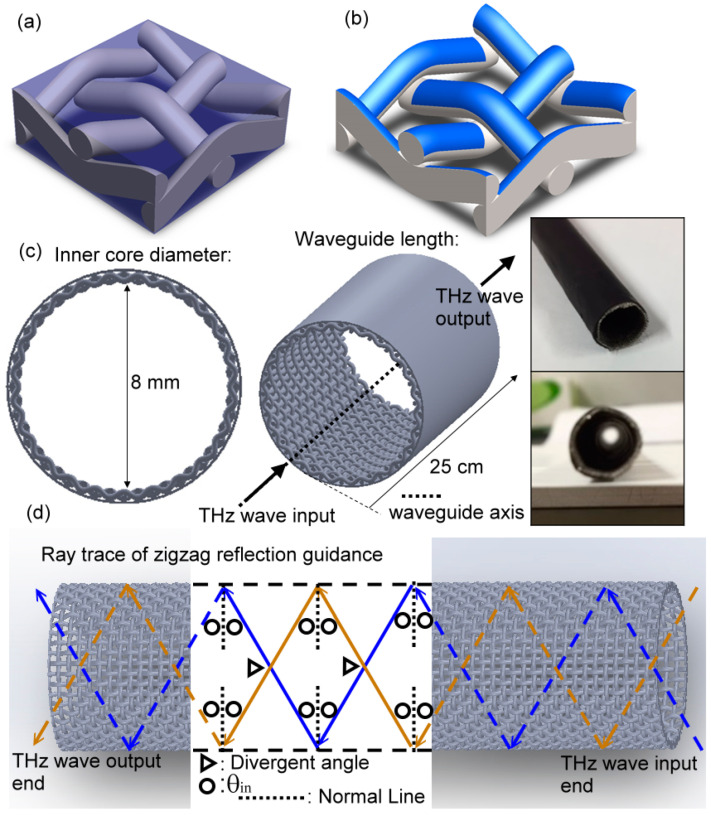
(**a**) Insulator integration of MWW-HA, (**b**) surface integration of the insulator and conductive layers on one side of MWW-HA, and (**c**) configuration of hollow-core pipe waveguide based on an MWW-HA. (**d**) Ray trace of zigzag reflection guidance of an MWW-HA pipe, where the dashed box indicates the specular reflection details at the MWW-HA pipe wall.

Given that the electric field of THz waves interacting with MWW-HA is tightly cumulated along the woven metal wires, the far-field imaging method of a camera cannot catch the electric field distribution on the corrugated metal wires, as shown in Figure 1d. To detect the THz electric field in a spectroscopic system, this study thus develops two integration methods for MWW-HA to modify the dielectric properties of metal holes [Figure 2a] and the surface conductivity of woven metal wires [Figure 2b].

To modify the air space of MWW-HA unit cells in Figure 1a–c, the insulator material of polyamic acid (PAA) was used to replace all the MWW-HA air space, as shown in Figure 2a. The PAA used in the experiment is in powder form, and it dissolves with an N-methyl pyrrolidone solvent (NMP). The PAA concentration, around 10–15 wt%, was adjusted for the suitable viscosity to adhere to the MWW-HA. In the adhesion process, MWW-HAs were soaked in the PAA solution, pulled up, and hung to drip excess PAA slowly. The integration of the PAA insulator with the MWW-HA [Figure 2a] was finally constructed after the NMP solvent volatilized. To perform the spectral variation of the MWW-HA THz field further, the PAA-integrated MWW-HA was, then, heated by an oven at 300 °C to react to imidization. From imidization, the PAA becomes polyimide (PI), which has a different molecular structure. The significance of the PAA and PI dielectrics for integrating MWW-HA is that they increase the OPL of the THz wave as it interacts with woven metal wires and their surrounding space.

To modify the surface conductivity of woven metal wires, the insulator and conductive layers were coated by a spatter machine on one side of MWW-HA [Figure 2b]. The material of this insulator layer is SiO_2_, and the prepared thickness is 500 nm. The conductive layer is made of aluminum-doped ZnO, denoted as Al:ZnO or AZO, with a thickness of 160 nm. The average resistivity of AZO is approximately 8 × 10^−2^ cm·Ohm. Figure 2b schematically shows the configuration of the nano-scaled thin film, integrating on an MWW-HA unit, without any dielectric filling in the structural space. The dielectric and conductive layers cover one-half of the surface areas of the woven metal wire surface, except for the sections under the upper woven metal wires. This surface integration approximates the exposing area of THz waves on an MWW-HA.

### 2.3. Hollow-Core Pipe Waveguide Based on MWW-HAs

To perform the specular-reflective THz wave guidance based on MWW-HA, one sheet of MWW-HA was deformed to one hollow-core pipe structure, as shown in Figure 2c. The deformation process is simply meant to cover the MWW-HA sheet on one brass metal rod surface, whose total length and diameter are, respectively, 30 cm and 8 mm. After tailoring the MWW-HA sheet area to exactly match the brass metal rod surface, one heat-shrink tube, having a 9 mm inner core diameter and being made of PE material, was then used to fix the deformed MWW-HA on the brass rod, which is operated by a heat gun to blow the PE tube. When the PE tube shrinks to fix the deformed MWW-HA sheet, the brass rod is removed from the fixed assembly to form a pipe wall. The combination of PE and MWW-HA tubes, constructing the outer and inner tube walls, constructs a hollow-core pipe waveguide for THz waves. The photos of Figure 2c express the end-face and three-dimensional views of an MWW-HA pipe waveguide, indicating the pipe wall compositions of a PE dielectric layer and an MWW-HA layer. The corresponding inner core diameter and transmission length are fixed at 8 mm and 25 cm, respectively, to observe the hollow core waveguide transmission, which is dependent on various MWW-HAs. The 8 mm inner core diameter is so large that THz radiation can easily be coupled without obvious losses at the input–end interface and from mismatching special modes. An EM wave transceiver in the THz spectrum can, therefore, be performed on the basis of an MWW-HA pipe waveguide.

### 2.4. Measured Parameters for MWW-HAs and MWW-HA Pipe Waveguides

To perform zigzag reflection guidance of a THz wave along an MWW-HA pipe, as schematically illustrated in Figure 2d, the bulk [13] and waveguide [14] schemes of THz time–domain spectroscopy (THz-TDS) were used to characterize MWW-HAs [Figure 1b] and their deformed pipe waveguide structures [Figure 2c], respectively. The beam spot range of input THz waves are 25 mm and 5 mm wide, respectively, for the MWW-HA material [Figure 1b and Figure 2a,b] and the hollow-core pipe waveguide [Figure 2c]. The ray trace of zigzag reflection guidance in Figure 2d indicates that the incident and reflected angles within the hollow core are identical, and it is denoted as *θ_in_* in Figure 1d. The divergence of the THz beam wave, denoted as triangles in Figure 2d, synchronously performs two parts of the zigzag reflection guidance on the pipe wall with a cylindrical symmetry. In theory, there are complex waveguide modes existing along this large hollow-core pipe waveguide, but only linear polarized waveguide modes can be detected from this waveguide scheme of THz-TDS that are constructed based on one pair of dipole antennas acting as a THz emitter and a detector [15,16].

The two THz wave parameters are power transmittance (*Tr.*) and phase retardation (∆*ϕ*), as measured in the two THz-TDS systems. Their definitions are shown in the following Equations (1) and (2).

(1) $$Tr.=\frac{P_{out}}{P_{in}}$$

(2) $$\Delta \phi =\frac{\phi_{out}-\phi_{in}}{2\pi }$$

$P_{in}$, $P_{out}$, $\phi_{in}$, and $\phi_{out}$ are individually denoted for the input and output THz waves’ powers and phases. They are obtained from the Fourier transform of THz electric field waveforms in the time domain, as measured in the THz-TDS. For the MWW-HA bulk material, a *Tr.* parameter represents the effective power loss at the MWW-HA structures, including the interfacial reflection, scattering loss, and any power depletion of roughness. The Δϕ parameter, measured for the MWW-HA bulk material, indicates the optical-path-length (OPL) change, time–domain waveform shift, or THz wave modulation on the MWW-HA structures. In this study, the aforementioned *Tr.* and Δϕ parameters of the MWW-HA bulk material are presented in the next section of results and discussion. The relating pipe structures, based on the MWW-HA bulk material, were measured only for the *Tr.* parameter in a waveguide scheme of THz-TDS [14] to identify the specular reflection of an MWW-HA pipe wall, which is also presented in the next section of results and discussion.

### 2.5. Theory of THz Wave Power Depletion and Reflection on MWW-HA Pipe-Wall Materials

For the illumination of broadband THz radiation on an MWW-HA, as shown in Figure 1d, the reflection, transmission, and surface-scattering performances are normally found based on the energy conservation of an optical material property. To guide THz radiation with specular reflection along a hollow-core MWW-HA pipe [Figure 2c], local electric field interacting MWW-HAs, especially, should be defined in theory for the mechanisms of power reflection and depletion, which present transmission spectral dips of MWW-HA plasmonic metamaterial. For linear polarized THz radiation in the bandwidth of 0.1–2 THz, the transmission spectrum can be characterized with metal hole transmission modes, which has the amplitude modulation of transverse resonance within a structural pore (*A*) and a unit-cell hole (2*A + D*) [14]. Given the symmetric metal hole structure, THz waves are supported by MWW-HAs with the mixed electric field between transverse electric (TE, ${\vec{E}}_{TE}$) and transverse magnetic (TM, ${\vec{E}}_{TM}$) waves, whose amplitudes are expressed in Equation (3).

(3) $$\left|{\vec{E}}_{MWW-HA}\right|=\int_{0}^{2D}\left|{\vec{E}}_{TE}\right|\cdot \left|{\vec{E}}_{TM}\right|dz$$

Given that TM waves have the longitudinal vector component of the electric field along the *Z* axis [Figure 1b], the amplitude loss of the THz MWW-HA wave, owing to the reflectance and surface scattering of power depletion, mainly results from the TE-wave vector component of ${\vec{E}}_{MWW-HA}$ (i.e., ${\vec{E}}_{TE}$) [14], whose direction of electric field oscillation is along the *X* axis [Figure 1b].

The wave function of the MWW-HA TE wave on the bulk MWW-HA structure [Figure 1b] (${\vec{E}}_{TE}$) is presented as the following Equation (4):

(4) $${{\vec{E}}_{TE}=E}_{x}\left(x,y,z,t\right)=\cos\left(2\pi x/\Lambda \right)\cdot \sin\left(m\pi y/A\right)\cdot \exp[i(2\pi \upsilon t-\beta z)],$$

where *m*, υ, and *β* are the integral numbers of resonant transmission modes, THz wave frequency, and the *Z*-axial propagation constant, respectively. The amplitude element of the wave function, $\cos\left(2\pi x/\Lambda \right),$ in Equation (4), is the modulation factor of metal-hole resonant transmission amplitude by periodically corrugated metal wires along the *X* axis (Figure 1) (i.e., the *X* axial woven metal wires). Even though the amplitude wave function of $\sin\left(m\pi y/A\right)$ presents transverse resonance inside the *A* aperture, the modulation part—$\cos\left(2\pi x/\Lambda \right)$—must be considered for the wave range covering the metal wire section in a *D* width. THz waves outside the metal hole of an *A* width or within the metal wire section in a *D* width can, thus, be modulated to perform noticeable power depletion, which presents the transmittance spectral dips. For the reflected THz waves from the bulk material of MWW-HA, their half wavelengths [*λ*, Equation (5)] should be larger than the sizes of MWW-HA pores (*A*) because of the following reality [14]:

(5) β<0, 2π/λ<mπ/A, A<λ/2

Equation (5) presents that the high-frequency-passed spectrum of plasmonic metamaterial for the MWW-HA [Figure 1b]), and the corresponding cut-off frequency with 10% power transmittance mimics the plasmonic frequency of metal [14]. Given the metal-hole rejection character, THz waves perform plasmonic reflection from MWW-HAs, whereas THz wave frequency is lower than the metal-like plasmonic frequency. It also means the MWW-HA-based plasmonic metamaterial can be used to reflect THz waves, specifically, in the low frequency band, where the highest frequency to cut off the high frequency band is defined from the criterion in Equation (5). The MWW-HA-dependent reflectivity for the interactive THz waves, especially, is contributed from the metal-hole *A* factor and the three-dimensional geometry of woven metal wires, including the factors of *D*, *A*, or *θ* (Table 1). Contrarily, the planar types of plasmonic metamaterials only have *D* and *A* parameters without the *θ* factor to control THz wave reflectivity. While optimizing the correlation among the geometry factors of *D*, *A*, or *θ*, the highest plasmonic reflectivity from an MWW-HA bulk material surface, corresponding to the specular reflection, can therefore be developed, in this study, as the pipe wall material to zigzag reflect THz waves that are lower than an MWW-HA cut-off frequency for a long distance and high waveguide transmittance.

## 3. Results and Discussion

**Figure 3:**
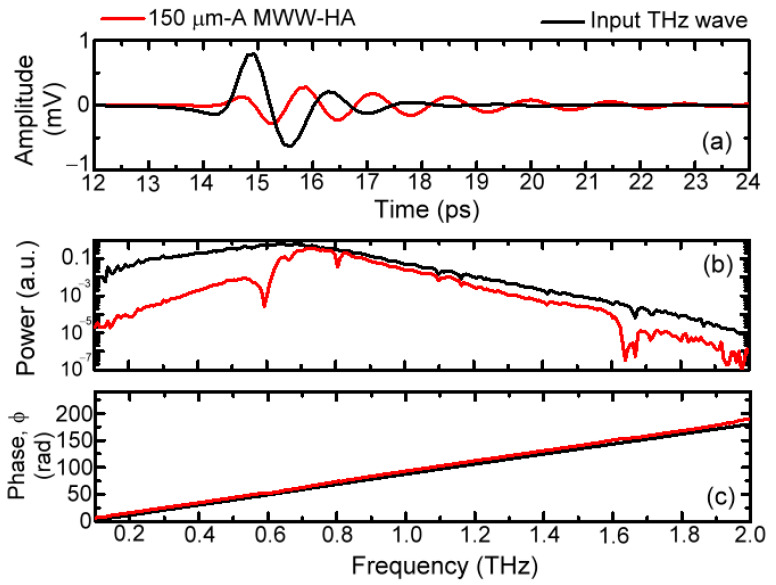
(**a**) THz time–domain waveforms measured in THz-TDS, as well as the relating (**b**) power transmission and (**c**) phase spectra.

**Figure 4:**
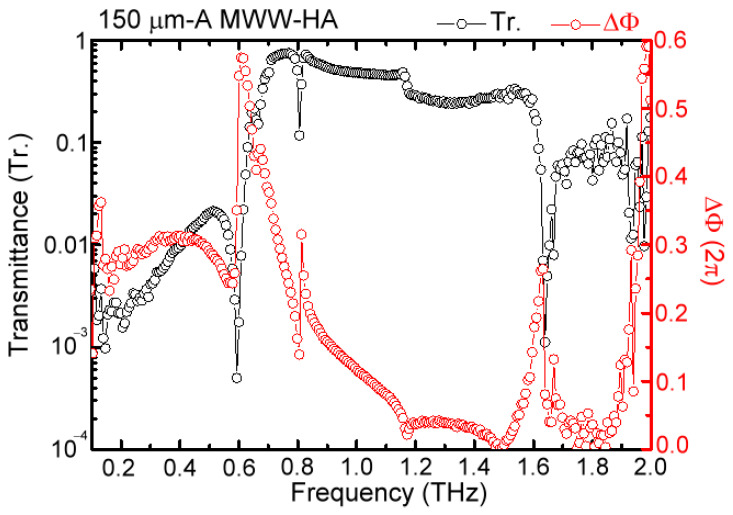
Power transmittance and phase retardation spectra of 150 μm-*A* MWW-HA.

Based on the three-dimensional schematic diagram of Figure 1b for measuring a transmission spectrum of MWW-HA plasmonic metamaterial, the THz waveforms of electric field oscillation in the time domain, illuminated on and output from one example of a 150 μm-*A* MWW-HA, are shown in Figure 3a. Figure 3a shows that the input THz pulse waveform out of the 150 μm-*A* MWW-HA is obviously broadened to shrink the input wave bandwidth, corresponding to filter out a certain spectral range of THz waves without transmission. Based on the Fourier–transform function of the MATLAB program, the corresponding power and phase spectra, individually shown in Figure 3b,c, were obtained. As THz frequency increases, the phase–spectral curve in Figure 3c is straightforwardly extended without winding, to constrain the maximum phase value of less than 2π radian, but it does not hinder the evaluation of measured ∆*ϕ* that was obtained from the propagation phase difference between the input and output MWW-HA THz waves. THz wave power loss is obvious for the transmitted power spectrum after passing this MWW-HA, and several sharp dips also occur. Based on Equations (1) and (2), transmittance (*Tr.*) and phase delay (or retardation, ∆*ϕ*) spectra of 150 μm-*A* MWW-HAs are, respectively, shown in Figure 4. The *Tr.* curve, denoted by a black circle line, has a high-frequency pass character with distinct spectral dips at 0.59, 0.805, 1.17, and 1.63 THz, which can be evaluated from the theory in Equation (4) for the MWW-HA TE mode. The spectral dip at 1.93 THz comes from the measurement deviation of the spectral edge, which is not reliable to be discussed in this study. The rejection band to reflect THz waves has a frequency of 0.1–0.59 THz. THz waves with high transmittance are in the frequency range of 0.59–1.63 THz, which becomes lower as the frequency is higher than 1.63 THz. For using the MWW-HA pipe wall material, the low-frequency-rejection waves (0.1–0.59 THz) can perform zigzag reflection transmission inside the hollow-core pipe. The ∆*ϕ* curve, denoted as a red circle line, shows spectral peaks around those *Tr.*-curve dips. For the transmitted bands in the frequency range of 0.59–1.48 THz, the trend of ∆*ϕ* curve performs an abnormal dispersion property. The average ∆*ϕ* value becomes the lowest value, corresponding to a low dispersion property, as THz wave frequency increases to 1.63–1.93 THz. These two dispersion properties, which were aforementioned, individually, at 0.59–1.63 THz and 1.63–1.93 THz, indicate the evanescent field transmission of MWW-HA emerging at the output end surface.

**Figure 5:**
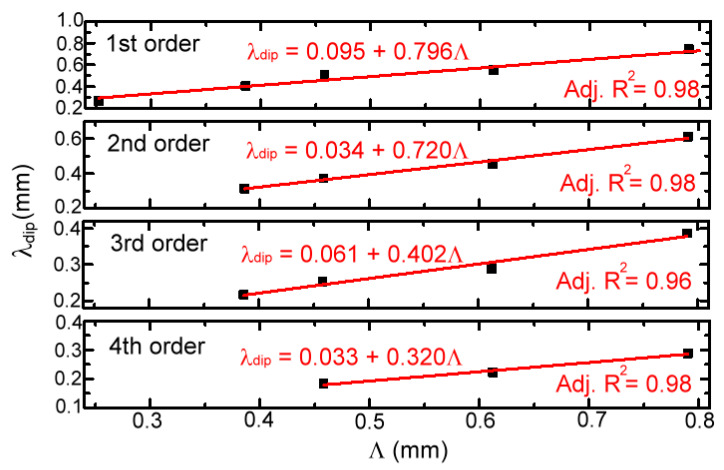
Multiple orders of power depletion measured in the transmittance spectral dips for the proposed MWW-HAs.

For various MWW-HAs, as shown in Table 1, their four spectral dips of *Tr.*-curves were further measured and correlated with corrugated periods (*Λ*) of woven metal wires in Figure 5. The spectral dips of MWW-HA at different frequencies resemble the wavelength or frequency-dependent power depletion of transverse resonance on the woven metal wires. Spectral dips within 0.1–2 THz have four orders, and the first spectral dip has the longest wavelength or the lowest frequency among the four orders of power depletion. The fitting curves between the spectral dip wavelength (*λ_dip_*) and MWW-HA period (*Λ*) were then obtained to study their power depletion mechanism on an MWW-HA surface. However, such a power depletion of spectral dips does not follow the resonance principle within a one-dimensional cavity of the corrugated woven metal wire. As shown in the evaluated slope of a fitting curve, *λ_dip_*/*Λ*, in Figure 5, the cavity width, *Λ*, is not related to the integer multiples of a half wavelength because the measured spectral dips do not follow the resonance principle that is controlled by the longitudinal wave phase, i.e., the $\exp[i\left(2\pi \upsilon t-\beta z\right)]$ part of Equation (4) [14], where the propagation constant β=0 makes 2π/λ=mπ/z, and z=mλ/2. The slopes of the first to the fourth spectral dips, with increasing THz wave frequency from 0.1 to 2 THz, are 0.796, 0.720, 0.402, and 0.320 for those MWW-HAs in Table 1. The side-view drawing in Figure 1c illustrates that one corrugated woven metal wire at the MWW-HA surface has one triangle-shaped cavity, but not a rectangular one, with a *Λ* base length (2A + 2D) to interrupt THz wave transmission. For the interrupted THz waves, the air space width to stay in the X axis is not uniformly equal to the *Λ* value while propagating along the Z axis. The power depletion of THz waves thus occurs for the Z axial propagation, which is interrupted on the metal woven wire surface and in the X-axial electric field (*E*-field) direction. Contrarily, those metal woven wire surfaces, perpendicular to the *E*-field (i.e., the Y axis), can pass the THz wave without any frequency-dependent power rejection or depletion of metal. From the transmission principle of metal holes in Equation (3), the TE mode of THz wave propagation, with those woven metal woven wires along the X-axial *E*-field, can deplete power owing to their corrugated shapes (i.e., a triangle-shaped cavity in the X-Z plane). As presented in Equation (4), the THz wave amplitude of TE mode transmission is modulated from the wave function of $\cos\left(2\pi x/\Lambda \right)$, owing to the corrugated metal woven wires in the *E*-field direction. The strength of the power depletion of MWW-HA, performing on the transmittance of spectral dips, is correlated with this amplitude wave function—$\cos\left(2\pi x/\Lambda \right)$—in theory [Equation (4)]. The four spectral dips in the experiment of Figure 5 also do not follow the regular diffraction orders of periodic metal holes or slits, and thus, we define the measured spectral dips of MWW-HA as the power depletion orders. As wave frequency increases from 0.1 to 2 THz, the first to fourth power depletion orders are respectively defined for the first to fourth spectral dips, as indicated in Figure 4 and Figure 5.

**Figure 6:**
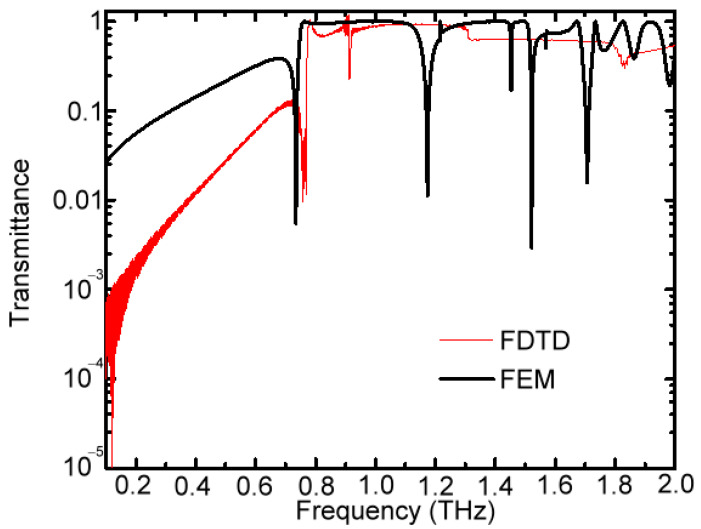
Theoretic transmittance spectra of 150 μm-*A* MWW-HA for the FDTD and FEM simulation methods.

For 150 μm-*A* MWW-HA, the distinct power depletion feature of transmission spectral dips in Figure 4 can also be found in the simulation of the finite-difference time–domain (FDTD) method in Figure 6, which was supported from the graphic and numeric functions of MATLAB programs. However, based on the reasonably chosen settings of a boundary condition, the finite element method (FEM) cannot predict these spectral dips well with those measured in the high-frequency-passed range [17]. The FEM method was also supported from the graphic and numeric functions of MATLAB programs. Figure 6 illustrates that the FEM simulation of the transmission spectrum at the high-frequency-passed range results in many noisy dips that are not observed in experiments (Figure 4), and they cannot be summarized with significant trends while tuning MWW-HA parameters (Table 1). The transmittance decay of around 1.8 THz, due to the increased resistance of resonant transmission, as shown at the measured result of Figure 4, is also found from the FDTD simulation (i.e., the red curve in Figure 6), not the FEM one. The increased resistance of resonant transmission basically comes from the middle woven metal wire in the unit cell structure [14]. FEM only simulates one structural unit of 150 μm-*A* MWW-HA, based on the Floquet analysis, to solve the integral formulation of Maxwell’s equations [17]. However, FDTD simulates the large structure reacting with the specified THz wave energy range in the experiment. That is, comparing the results between FEM and FDTD simulation methods, as shown in Figure 6, presents that the used MWW-HA structure should cover the transverse beam spot range of input THz waves, not only one-unit cells [Figure 1b,c and Figure 2a,b].

**Figure 7:**
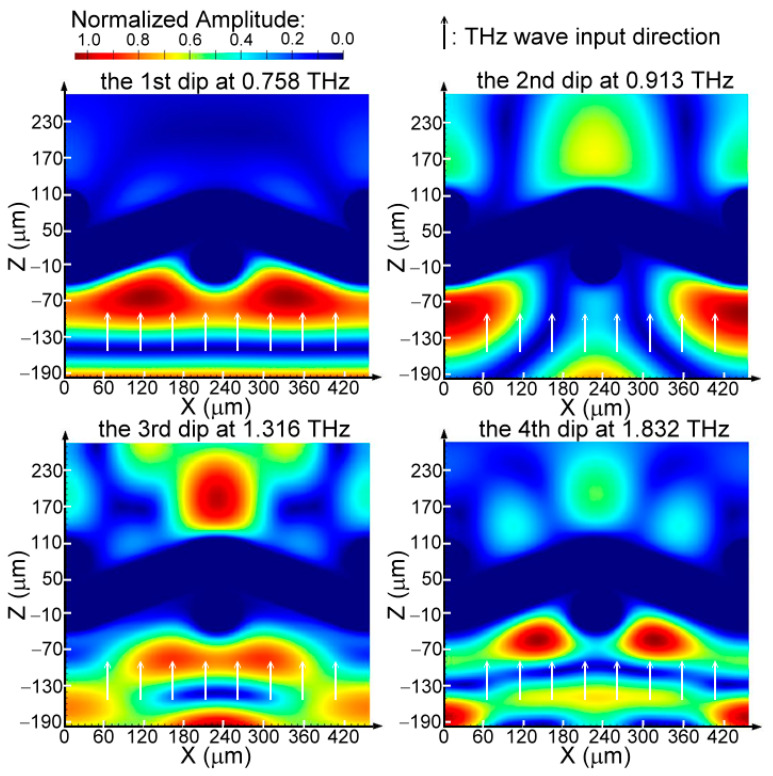
Electric field distribution, in theory, for multiple orders of power depletion on the X-axial woven metal wires.

Among the measured transmission spectral dips in Figure 4, the third order of power depletion at 1.17 THz is the lowest, which is also found in the calculation of the FDTD simulation (i.e., the red curve in Figure 6). Based on the amplitude modulation part of X-axial woven metal wires, $\cos\left(2\pi x/\Lambda \right)$ [Equation (4)], and the measured slope of *λ_dip_*-*Λ* relation in Figure 5 (0.796, 0.720, 0.402, and 0.320), the responding amplitudes of 150 μm-*A* MWW-HA to transmit THz waves are approximately $\cos\left(1.592\pi \right)$, $\cos\left(1.44\pi \right)$, $\cos\left(0.804\pi \right)$, and $\cos\left(0.64\pi \right)$. The 150 μm-*A* MWW-HA transmitted wave at the third order of power depletion has the highest absolute amplitude value, 0.81634 [i.e., $\cos\left(0.804\pi \right)$], and therefore the spectral dip is especially unclear. Such consistence of spectral-dip visibility between the measurement (Figure 4) and the FDTD simulation (Figure 6) is also found in the strong power depletion at the other three spectral dips in 0.1–2 THz (i.e., the first, second, and fourth power depletion orders). Furthermore, the curve feature of high-frequency-passed and plasmonic reflection spectra, aside from one cut-off plasmonic frequency, is presented by the FDTD simulation (Figure 6), which is also consistent to that of the measured result in Figure 4 and the Equation (5) definition. Consequently, the normalized amplitude of *E*-field distribution on the X–Z plane of the Y-axial center (Figure 1) is further calculated by the FDTD method for the 150 μm-*A* MWW-HA, which is illustrated in Figure 7. The third spectral dip in Figure 7 has the strongest *E*-field at the output end face, which is compared among the *E*-field distributions of the four spectral dips in the X–Z plane. At the input end (z = −70–−10 μm, Figure 7), *E*-field cumulates on the woven metal wire of the 150 μm-*A* MWW-HA unit. As the THz frequency of a spectral dip increases from the first to the fourth order dips of power depletion, the slope value of *λ_dip_*/*Λ* reduces (Figure 5) to move those field cumulations of metal surface waves at z = −70–−10 μm for approaching the middle-woven metal wire section, which locates approximately 240 μm of the X axis (Figure 7).

The frequency of the first-order power depletion is located in the spectral range of metal-hole rejection waves [Equation (5)], as shown in Figure 4 and Figure 6. Thus, this character of the strongest power depletion dominates THz wave reflection behavior on the MWW-HA surface. Given that the MWW-HA is not a planar structure, two parameters influence THz wave reflection and transmission performance, including the OPL and metal surface conductivity of the woven metal wires. The corresponding MWW-HA configurations to change OPL and metal surface conductivity are, respectively, illustrated in Figure 2a,b. For integrating PI and PAA polymers with an MWW-HA [Figure 2a], the refractive indices of these two polymer dielectrics are higher than that of air, 1.0, and they can increase OPL of MWW-HA, which can correspond to the condition of *θ_in_* > 0° to reflect THz waves from blank MWW-HAs.

**Figure 8:**
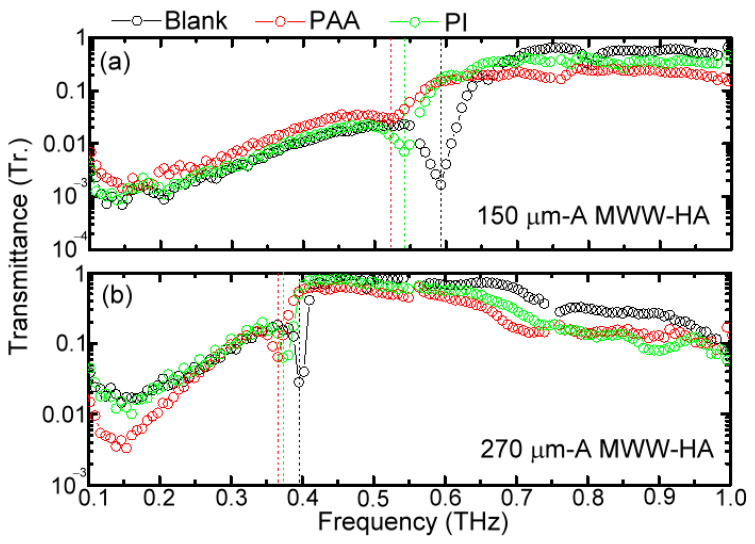
Power transmittance spectra measured for the integration of PI and PAA insulators with (**a**) 150 μm and (**b**) 270 μm-*A* MWW-HAs.

Figure 8a,b, respectively, present the transmittance spectral variations of 150 μm and 270 μm-*A* MWW-HAs that were integrated by PI and PAA polymers. The high-frequency wave passing–transmittance curves for integrating PI and PAA dielectrics can be preserved to perform a redshift at the first spectral dip and cut-off frequencies of MWW-HA, which define the spectral range of low THz frequency for reflecting waves. The spectral shift range, compared with that of a blank MWW-HA, is proportional to the THz refractive index or wavenumber surrounding the corrugated space of woven metal wires [Equations (3)–(5)]. The X and Y-axial amplitudes of the MWW-HA TE wave function in Equation (4) change and are shown as ${{\vec{E}}_{TE}=E}_{x}\left(x,y,z,t\right)=\cos\left(2\pi n_{eff}x/\Lambda \right)\cdot \sin\left(mn_{eff}\pi y/A\right)\cdot exp[i(2\pi \upsilon t-\beta z)]$, where *n_eff_* indicates the effective refractive index of THz waves on MWW-HAs. The measured spectral ranges of redshift in Figure 8a,b show that the THz refractive index of PAA is higher than PI, owing to high molecular polarity. The thermal process of imidization only removes the water element, H_2_O, from a PAA molecule to become the PI molecule. The molecular polarity of H_2_O is high, making PAA molecules perturbed easily by THz waves compared to PI molecules. It also means the time-dependent electric field of THz waves in PAA is more delayed than in PI. Furthermore, the increased OPL condition weakens power depletion with increased transmittance at the first spectral dip of MWW-HA [Figure 8a,b]. Therefore, the *E*-field on the woven metal wire surface partially distributes inside and outside the PAA or PI dielectric [Figure 2a], consequently reducing the strength of power depletion from the woven metal wires. However, for the increased OPL, only the first-order dip of power depletion can be preserved, and the other high-order dips disappear with rising transmittance.

**Figure 9:**
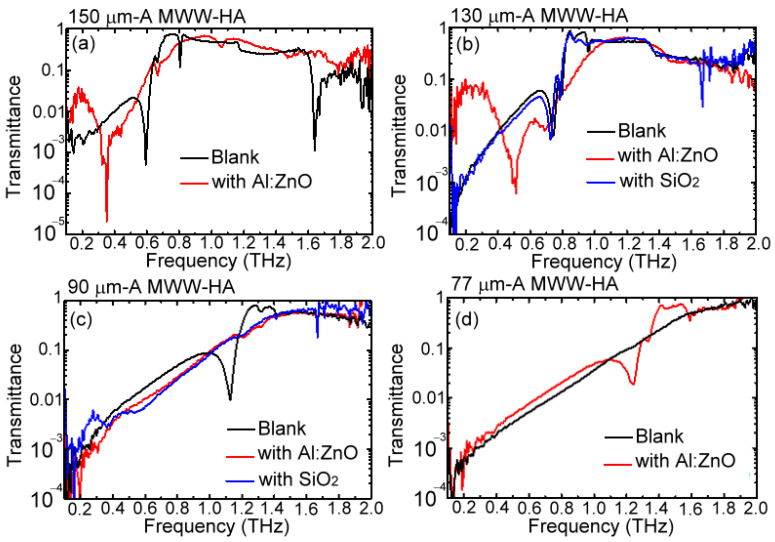
Power transmittance spectra of (**a**) 150 μm, (**b**) 130 μm, (**c**) 90 μm, and (**d**) 77 μm-*A* MWW-HAs for the surface layer integration of AZO and SiO_2_.

The woven metal wires in the study are made of stainless steel, and they can be considered perfect conductors. To reduce the conductivity of woven metal wires to interact with THz waves, the insulator and conductive layers were, respectively, applied from SiO_2_ and AZO to coat one side surface of an MWW-HA [Figure 2b]. In the experiment, four MWW-HAs, including the *A* parameters of 150, 130, 90, and 77 μm, were coated with 160 nm-thick AZO, and their measured transmittance spectra in 0.1–2 THz are individually illustrated in Figure 9a–d.

The blank 130 μm and 90 μm-*A* MWW-HAs were coated by a 500 nm-thick SiO_2_ film on one side surface of a blank MWW-HA; their measured transmittance spectra are also expressed in Figure 9b,c, and they are denoted by the blue spectral curves. For the AZO-coated woven metal wires, the conductivity largely reduces, and the measured frequencies of the first spectral dips redshift, as presented on the 150 μm and 130 μm-*A* MWW-HAs [the red spectral cures in Figure 9a,b]. However, for the approximate amplitude modulation $\cos\left(2\pi x/\Lambda \right)$ between the first and fourth spectral dips of 150 μm-*A* MWW-HA [Figure 9a], the fourth spectral dip at 1.63 THz flattened without any spectral shift effect. Although the 160 nm-thick AZO and 500 nm-thick SiO_2_ have approximate OPL, considering their refractive indices in THz frequency and their physical thickness, their redshift at the first spectral dip is different, as shown in Figure 9b. Figure 9b expresses that the SiO_2_-coated surface almost cannot shift the first spectral dip of 130 μm-*A* MWW-HA, but a very large redshift occurs when the woven metal wires are coated with the AZO layer. However, the first-order dip of the 90 μm-*A* MWW-HA at 1.127 THz disappears, as shown in Figure 9c, while coated with AZO and SiO_2_ layers. Figure 9d shows that the 77 μm-*A* MWW-HA, originally, does not have any transmittance spectral dip in the 0.1–2 THz range, but the AZO-coated surface contrarily induces one transmittance spectral dip at 1.23 THz, which is the first-order dip for the MWW-HA power depletion.

Comparing the MWW-HA geometry in Table 1, the 90 μm-*A* MWW-HA has the smallest bending angle *θ* of woven metal wires: 36.22°. Based on the investigation of power depletion at the first spectral dip in Figure 4, Figure 5, Figure 6 and Figure 7, the *θ* factor of woven metal wires is critical in spectral dip generation. The smallest *θ* of 90 μm-*A* MWW-HA is, thus, the reason why AZO and SiO_2_-coated layers can eliminate the power depletion of woven metal wires [Figure 9c]. As the woven metal wire bending angle *θ* increases from 36.22° to 40.85° for the case of 77 μm-*A* MWW-HA, the conductive AZO layer can generate the spectral dip at 1.23 THz [Figure 9d]. For the case of 130 μm-*A* MWW-HA with a larger metal wire bending angle, *θ* = 44.61°, the SiO_2_ insulator surface preserves the spectral dip of power depletion on woven metal wires [Figure 9b], and the variation is not obvious. On the contrary, the AZO conductive layer on 130 μm-*A* MWW-HA preserves the first-order power depletion’s spectral dip and redshifts the spectral dip to a relatively low frequency with a frequency range of 0.227 THz [Figure 9b]. The same AZO layer on the 150 μm-*A* MWW-HA performs a stronger redshift with a frequency range of 0.241 THz [Figure 9a], which is compared with that of 130 μm-*A* MWW-HA [Figure 9b]. The geometry comparison between 130 μm and 150 μm-*A* MWW-HAs in Table 1 shows that the larger woven wire bending angle, *θ =* 48.29° > 44.61°, results in the larger redshift range, owing to the larger effective refractive index (*n_eff_*) of an MWW-HA OPL, even though they are coated with the same 160 nm-thick AZO layer. The *n_eff_*, obtained from the propagation constant *β*, fundamentally correlates with the phase retardation [∆*ϕ*, Equation (2)] of MWW-HA THz waves based on the dielectric dispersion principle, $n_{eff}=1+\left[C\Delta \phi /\left(2\pi \upsilon \cdot {\Delta Z}_{eff}\right)\right]$, where the ∆*Z_eff_*, υ, and *C* parameters individually represent effective thicknesses of MWW-HA for interacting with THz waves, THz wave frequency, and propagation speed in a free space. On the same woven metal wires of 150 μm-*A* MWW-HA, the power depletion at the fourth order of the power depletion is easily eliminated without redshift by the surface modification of the 160 nm-thick AZO layer [Figure 9a]. Given that the cumulated field location at the fourth dip approaches the middle-woven metal wire at 240 μm of X axis [Figure 7], the TM modal field based on the Y-axial metal wire array enhances due to the added AZO layer to pass the metal holes instead of the TE field reflection from the X-axial metal wire array.

**Figure 10:**
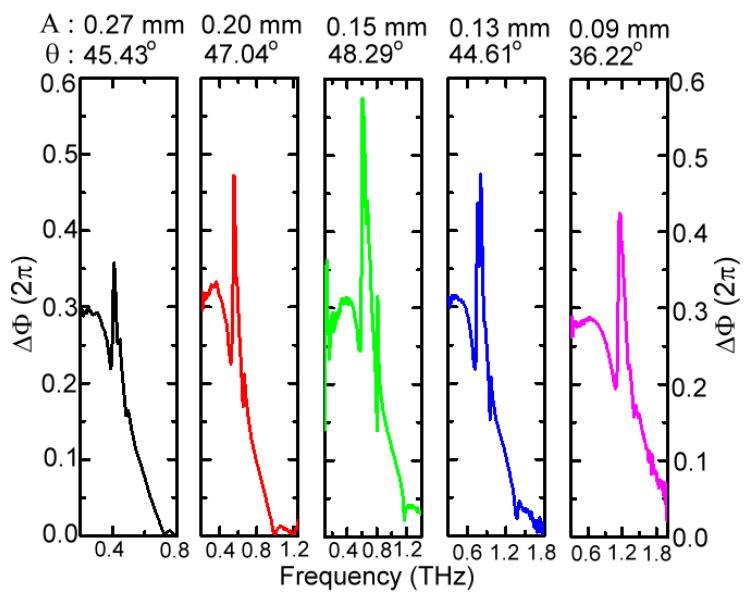
Phase retardation spectra of different MWW-HAs, and the structural parameters of *A* and *θ* for each MWW-HA.

**Figure 11:**
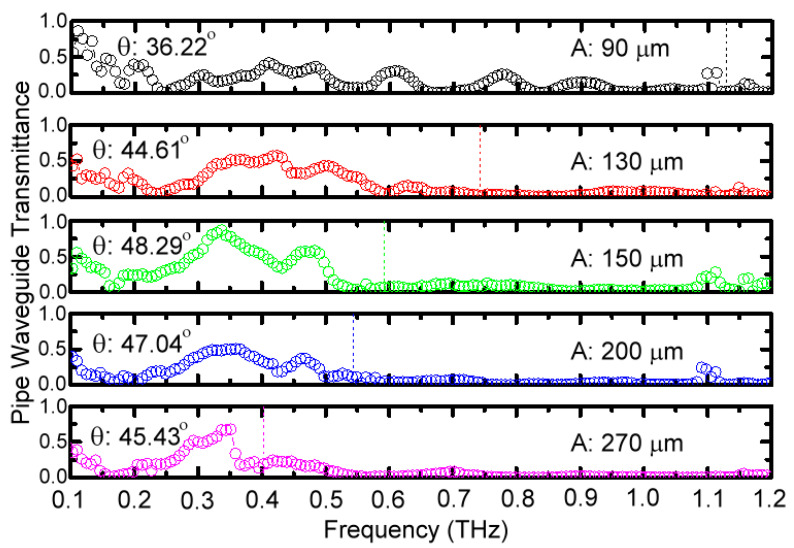
Transmittance spectra of pipe waveguides based on different pipe wall materials of MWW-HAs.

The ∆*ϕ* spectrum of 150 μm-*A* MWW-HA in Figure 4 is replotted in Figure 10 and compared with other MWW-HAs in Table 1 with 0.27, 0.20, 0.13, and 0.09 μm-*A* values. The 150 μm-*A* MWW-HA has the highest ∆*ϕ* response around the first spectral dip frequency, and the ∆*ϕ* value peak approximates to π and occurs in the frequency range of a metal hole reflection band. Such a π-∆*ϕ* response of the 150 μm-*A* MWW-HA indicates that the performance of specular reflection can be obtained based on the 48.29°-*θ* woven metal wires and MWW-HA structure (Figure 1 and Table 1). The π retardation of metal holes certainly cannot be achieved by the planar metal hole arrays that are perforated from one metal slab because of the zero longitudinal space in an optic axis to modify the THz wave propagation constant. For presenting this character of specular reflection guidance, based on the 150 μm-*A* MWW-HA, a hollow-core pipe configuration, as shown in Figure 2c, is used to control and reflect THz waves to guide straightforwardly in a 25 cm-long distance. Given that the large core of an 8 mm diameter is much larger than the wavelengths for 0.1–1 THz waves (0.3–3 mm), THz waves zigzag reflect from the MWW-HA pipe wall. Figure 11 shows the transmittance of hollow-core pipe waveguides based on various MWW-HAs, as shown in Table 1, as well as the configuration of Figure 2c, where the dotted lines indicate the measured THz frequencies of the first spectral dips, owing to the power depletion on the woven metal wires [Figure 5]. The measured and compared results of Figure 11 show that the 150 μm-*A* MWW-HA with the largest *θ* value, 48.29°, performs the highest transmittance of a 25 cm-long hollow-core pipe waveguide, and the corresponding transmittance spectral peak is up to 0.869 at 0.336 THz. As the *θ* parameter of metal woven wire reduces from 48.29° to 47.04° (200 μm-*A* MWW-HA) and 45.43° (270 μm-*A* MWW-HA), the corresponding transmittance peak of the hollow-core pipe reduces to 0.504 and 0.676 at 0.358 and 0.351 THz, respectively. Consistently, when further reducing the *θ* parameter of MWW-HA from 48.29° to 44.61°, the corresponding transmittance peak of the hollow-core pipe reduces to 0.574 and locates at 0.424 THz. Therefore, among these MWW-HA pipe wall materials (Table 1), the ∆*ϕ* spectral peak of π for the 150 μm-*A* MWW-HA (Figure 10) exactly realizes the highest reflectivity within its plasmonic reflection band [<0.65 THz, Figure 4 and Equation (5)] that approaches specular reflection inside the MWW-HA-pipe core [Figure 2c] because of its largest metal wire bending angle *θ* [Figure 1b]. This specular reflection of the 150 μm-*A* MWW-HA pipe wall eventually performs the highest transmittance, up to 0.869, for a 25 cm-long pipe length. The pipe-transmission spectral peak is located at 0.336 THz, but it is lower than the frequency of the first power depletion order, 0.59 THz (Figure 4), because of the increased OPL [Figure 8a and Figure 9a] of the zigzag reflection process [*θ_in_* ≠ 0°, Figure 1d]. For the 90 μm-*A* MWW-HA material, a pipe waveguide transmittance peak of 0.862 is at the spectral edge, 0.1098 THz, owing to the smallest banding angle of woven metal wire (*θ* = 36.22°, Table 1). It effectively comes from the power reflection of a planner metal surface, not that of periodically corrugated metal wires (i.e., the woven metal wires of an MWW-HA structure). It normally occurs for the extremely low-frequency waves without correlating to the three-dimensional structure of MWW-HA, including the geometric factors of *θ*, *A*, and *D*. In the frequency range of 0.15–1.13 THz, those metal holes of 90 μm-*A* MWW-HA are too small to leak power from the pipe core based on the plasmonic reflection criterion at Equation (5), A<λ/2, but the corresponding 36.22°-*θ* value is too small to deviate the specular reflection (∆*ϕ* = π) performance at 48.29°-*θ*. When the pipe-guided THz wave frequency further reduces down to approximately 0.1 THz, the *θ* factor disappears without retardation on the THz wave phase. It means the corresponding wavelength is much larger than the cross-section of woven metal wires without Z-axial longitudinal interaction. Only the *A* factor contributes specular reflection within the 90 μm-*A* MWW-HA pipe for the transmittance spectral peak.

## 4. Conclusions

To realize specular reflection along a 25 cm-long hollow-core-pipe wave guidance, metal hole-reflected THz waves with phase optimization were experimentally demonstrated based on the MWW-HA structure. The periodically corrugated construction of woven metal wires performs strong phase retardation as one spectral peak in 0.1–2 THz that can be characterized by the bending angle parameter *θ*. For the unit structure of MWW-HA, the corrugation of woven metal wires along the electric field, called the TE modal field, specifically modulates the MWW-HA THz wave amplitude with noticeable power depletion by the factor of $\cos\left(2\pi x/\Lambda \right)$. In the study, the specified power depletion of the MWW-HA TE modal field performs four orders of spectral dips in the transmittance spectrum of 0.1–2 THz. However, only the first order of power depletion at the metal-hole reflected band and with a sufficiently large bending angle of woven metal wires, *θ* > 40°, can be preserved for increasing OPL of MWW-HA THz waves. The 48.29°-*θ* MWW-HA is composed of a pore size (*A*) of 150 μm, and a metal wire width (*D*) of 79 μm presents the highest phase retardation of metal-hole reflected waves up to approximately π, corresponding to the highest efficiency of reflection as specular reflection. Based on the deformable property of the MWW-HA, a 0.336 THz wave zigzag reflects along a hollow-core pipe with a 48.29°-*θ* MWW-HA surface, whose inner core diameter and cylindrical length are, respectively, 8 mm and 25 cm, and the measured waveguide transmittance reaches 0.869. Such high efficiency of metal-hole-array reflected THz wave guidance is, thus, specialized from the three-dimensional structure of woven metal wires instead of the two-dimensional plane of the metal-hole array. The MWW-HA-based plasmonic metamaterial is, therefore, the novel artificial material in the THz frequency band because it not only performs plasmonic reflection, such as the available planar plasmonic metamaterials, but it also modulates the reflected THz wave phase for the highest reflectivity that is not presented yet.

## Figures and Tables

**Table 1:** Geometry parameters of MWW-HAs.

Parameters	Structure Number of MWW-HA					
	1	2	3	4	5	6
A (μm)	270	200	150	130	90	77
D (μm)	125	106	79	63	36	35
θ (°)	45.43	47.04	48.29	44.61	36.22	40.85

## Data Availability

Not applicable.
